# Low-Grade Appendiceal Mucinous Neoplasm Causing Equivocal Appendicitis: A Case Report

**DOI:** 10.7759/cureus.56286

**Published:** 2024-03-16

**Authors:** Evan J Gorgas, Shawn C Dowling

**Affiliations:** 1 General Surgery, Trinity Health Ann Arbor Hospital, Ypsilanti, USA

**Keywords:** low-grade appendiceal mucinous neoplasm, laparoscopic appendectomy, pelvic congestion syndrome, diagnostic laparoscopy, atypical appendicitis

## Abstract

Appendicitis is one of the most common diagnoses that general surgeons encounter in practice. An exceedingly rare cause of this disease is neoplasm. We report the case of a 24-year-old female who presented with non-specific right lower quadrant abdominal pain and equivocal findings of appendicitis and pelvic congestion syndrome on CT imaging. After an extensive work-up, the patient underwent a diagnostic laparoscopy with an appendectomy. The appendix appeared grossly normal; however, on a pathologic review of the specimen, a low-grade appendiceal mucinous neoplasm (LAMN) was found. This case is unique in that it demonstrates exclusive management of LAMN laparoscopically. It reinforces the need to approach non-specific abdominal pain from a multidisciplinary perspective and to utilize laparoscopy as a diagnostic/therapeutic modality when other, less invasive, modalities fail to diagnose a patient’s pain.

## Introduction

Low-grade appendiceal mucinous neoplasm (LAMN) is a rare neoplastic finding with a reported rate between 0.4 and 1.0% of all appendiceal specimens [1]. Patients present with clinical findings consistent with acute appendicitis secondary to the luminal obstruction of the lesion, or it will be incidentally found during work-up for vague abdominal pain [2-3]. Cross-sectional CT imaging may reveal a tubular cystic structure, calcifications in the wall of the appendix, a dilated lumen > 1.3 cm, low contrast attenuation, irregular wall thickening, and likely no evidence of surrounding inflammation [4-5]. Furthermore, if not incidentally found on pre-operative imaging, more are identified incidentally during operative exploration or on histopathologic analysis post-operatively [2]. Without evidence of metastatic disease, these lesions can often be managed with appendectomy alone [2]. In the following, we report the case of a 24-year-old female with an acute onset of non-specific right lower quadrant abdominal pain and equivocal CT findings of appendicitis. After a multi-disciplinary work-up for her abdominal pain, she ultimately underwent diagnostic laparoscopy with appendectomy and was incidentally found to have a LAMN. This case demonstrates the importance of maintaining a broad differential in the work-up of abdominal pain, the management of incidentally found LAMN, and laparoscopy as an important diagnostic/therapeutic modality in the treatment of non-specific abdominal pain.

## Case presentation

The patient is a 24-year-old woman with a history of irritable bowel syndrome (IBS) and asthma who presented to the ED with the acute onset of right lower quadrant abdominal pain. Her pain started the morning of her presentation and was exacerbated with movement. She had associated anorexia and nausea, but no vomiting. She reported an elevated temperature at home of 100°F. Bowel movements were of a liquid consistency, which she reported was normal for her. Her last menstrual period was about two weeks prior to the presentation. She reported that her menstrual periods can be associated with significant pain. She initially thought she was having an IBS flare or UTI, as her pain was similar in quality. However, when the pain intensified, she presented for further evaluation. Finally, she reported that a few weeks prior to the presentation, she had a similar episode of pain that resolved spontaneously after three days.

**Figure 1:**
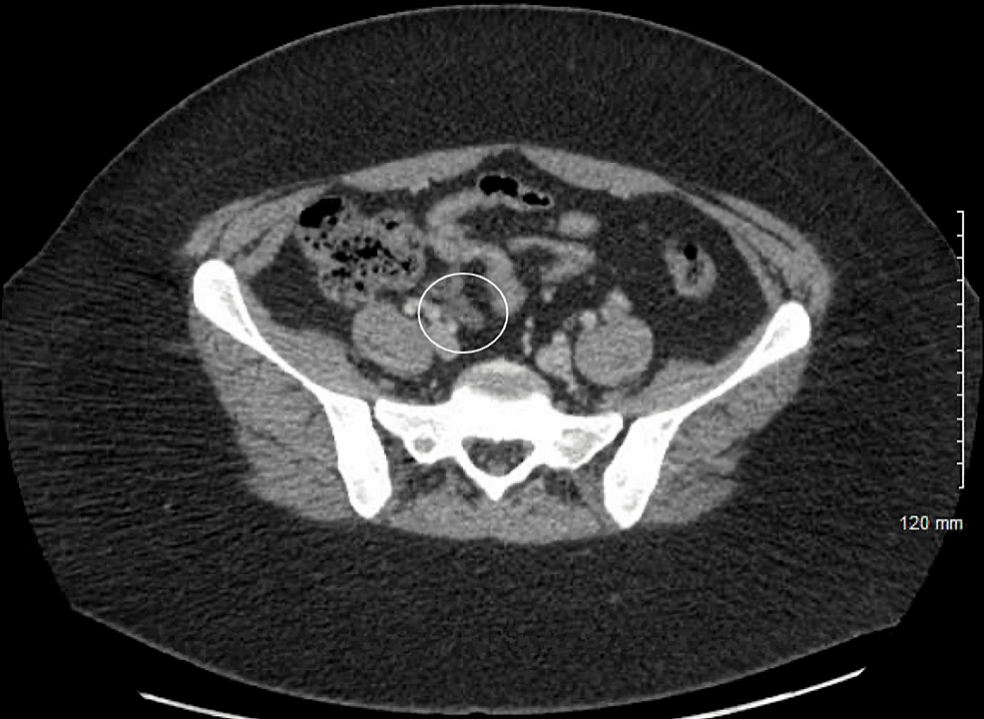
Admission CT scan Axial CT with IV contrast demonstrates a mildly dilated appendix encircled above. CT: computed tomography; IV: intravenous

In the ED, her vitals were within normal limits. Her BMI was 42. Her abdomen was noted to be soft, non-distended, and tender in the right lower quadrant without rebound or guarding. Her labs, including a complete blood count, comprehensive metabolic panel, lipase, beta-human chorionic gonadotropin, and urinalysis, revealed no abnormalities; all values were within the normal range. Given her symptoms and physical exam findings, a CT of the abdomen and pelvis with intravenous (IV) contrast was ordered prior to surgical consultation and demonstrated a mildly dilated appendix to 0.9 cm with intraluminal fluid and air but no wall thickening or peri-appendiceal inflammation (Figure 1).

With regard to the gynecologic sources of her pain, the CT showed no ovarian or uterine lesions. However, the right gonadal, uterine, and adnexal veins were equivocally congested, raising concern for possible pelvic congestion syndrome (PCS). In the ED, she continued to have pain and was thus admitted to the general surgery service for observation (without antibiotic therapy) and further diagnostic work-up prior to operative intervention.

On the first day of her hospitalization, a transvaginal ultrasound was obtained and showed nonspecific pelvic venous congestion but no other concerning findings. She continued to remain afebrile, without leukocytosis, and without other lab abnormalities. However, her pain continued to worsen, and gynecology was consulted. She denied any symptoms that would raise concern for sexually transmitted infections (STIs) or pelvic inflammatory disease. On a bimanual pelvic exam, she had right adnexal tenderness but otherwise no other concerning features. Given the acute nature of her pain, they did not favor a diagnosis of PCS, as it typically presents with chronic pain. Her pain continued to persist, and on day 3, repeat imaging was obtained. As there was concern for worsening appendicitis, a CT with IV, oral, and rectal contrast was obtained that demonstrated no new or worsening findings that would explain her pain. She continued to remain afebrile, hemodynamically normal, and without lab abnormalities.

On day 4, her pain continued, and the patient consented to undergo a diagnostic laparoscopy with a likely appendectomy. The attending surgeon on-call performed her operation. During the consent process, the patient was made aware that her pain may not resolve with the operation, and she agreed to proceed despite this. Gynecology was aware we were taking the patient to the operating room and would be available if needed. In the operating room, the stomach, small bowel, colon, uterus, and ovaries appeared normal. Notably, the appendix also appeared grossly normal. The appendectomy proceeded in the usual fashion, and the patient was discharged following the procedure. The appendix was sent to pathology for a permanent section.

**Figure 2:**
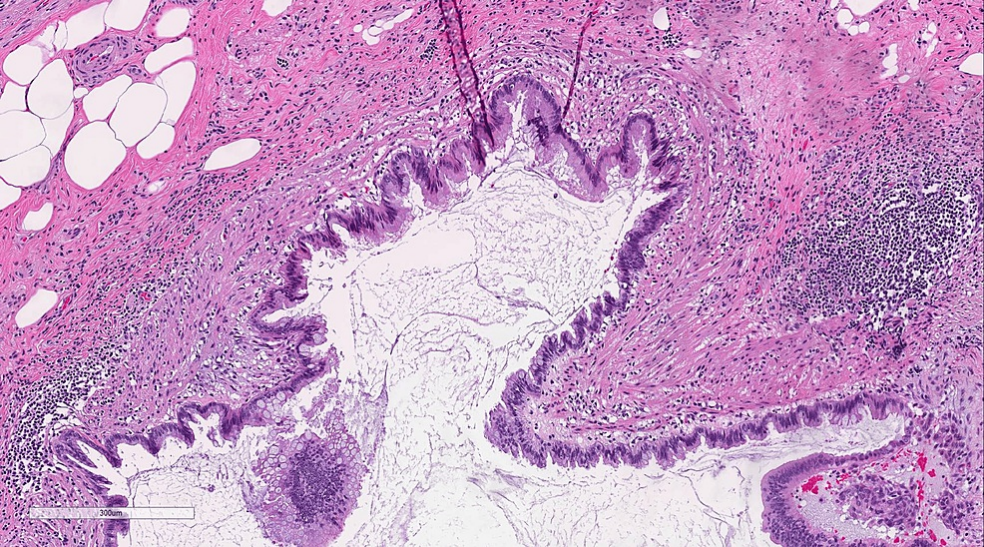
Representative cross-section of LAMN LAMN at the tip of the appendix. Acellular mucin is visualized in the lumen of the appendix. Low-grade dysplasia is seen in the neoplastic epithelial lining with nuclear pseudostratification. LAMN: low-grade appendiceal mucinous neoplasm

On postoperative day 4, the patient’s surgical pathology resulted in a margin-free, well-differentiated LAMN (Figure 2). The tumor was on the distal aspect of the appendix, and the appendix showed no sign of perforation. There was marked dilation of the distal lumen, with translucent mucoid material confined to the appendiceal wall. Given these findings, the patient was scheduled for a screening colonoscopy six weeks post-operatively and follow-up with medical oncology. At her post-operative clinic follow-up appointment, she reported that her pain had completely resolved. Carcinoembryonic antigen and cancer antigen-125 were also found to be normal.

## Discussion

Appendicitis remains the most common reason to undergo emergency abdominal surgery, with a reported rate of 96.5-100 per 100,000 adults annually [6]. Appendicitis is ultimately caused by obstruction of the appendiceal orifice by means of an appendicolith, lymphoid hyperplasia, or neoplasm [6]. History, physical and laboratory work, and imaging are essential in the diagnosis of appendicitis, especially in pre-menopausal women who may have a gynecologic etiology to their pain. Patients usually present with fever, leukocytosis (white blood cell count greater than 10,000/µL), and CT findings consistent with appendicitis (dilation > 7 mm, wall thickening, periappendiceal fat stranding, abscess, phlegmon, etc.) [6]. Notably, if there is air visualized in the lumen, appendicitis can likely be ruled out given that the presence of air eliminates an obstructive process. As reported, our patient did not demonstrate clear findings of appendicitis during her hospitalization, and thus further diagnostic work-up was needed to identify the cause of her symptoms.

After ruling out pregnancy, UTI, STIs (studies ultimately did not reveal any infection), and adnexal/uterine mass, there was concern for PCS. Patients with this condition are typically pre-menopausal and present with vague, chronic sensations of pelvic pain, heaviness, or fullness for several months. Symptoms can be exacerbated with activity, menstruation, pregnancy, and intercourse. The first step in diagnosis is often pelvic ultrasound, which may show dilated ovarian veins > 4 mm, a tortuous venous plexus, slowed or retrograde blood flow, or polycystic ovarian changes that differ from typical polycystic ovarian syndrome [7]. It is important to note that these findings are non-specific. Again, this patient had equivocal findings of PCS, and after conferring with gynecology, we were comfortable eliminating a gynecologic source of her pain.

Laparoscopy remains an important diagnostic and therapeutic tool in the management of nonspecific abdominal pain. When physical exam, laboratory, and radiologic findings do not clearly determine a source of pain, laparoscopy is an important adjunct to consider. In hemodynamically stable patients without clear evidence of needing emergent laparotomy, early diagnostic laparoscopy has been shown to prevent delay in treatment, prevent unnecessary laparotomy, and reduce hospital length of stay [8]. As our patient continued to have significant pain, had multiple non-diagnostic, non-invasive imaging modalities, and had no laboratory or hemodynamic abnormalities, diagnostic laparoscopy was the best next step in the management of her symptoms.

As previously stated, LAMN is a rare neoplastic finding on appendectomy. Incidentally found lesions that are less than 2 cm, do not involve the mesoappendix or appendiceal base, do not show signs of perforation, and without evidence of mucinous ascites can be managed with laparoscopic appendectomy alone [2]. If there is concern that the appendix cannot be removed without causing perforation, it is recommended to extend trochar incisions or convert to laparotomy [3]. If the lesion involves the mesoappendix, base, or is > 2 cm, a right hemicolectomy is recommended [2]. Our patient had a grossly normal-appearing appendix; the lesion was 3 cm away from the base and was margin-free on histopathologic review, and thus no further surgical intervention was needed. In reviewing the literature, there are reports of incidentally found LAMNs requiring open intervention, but few are managed with laparoscopy alone [1,5,9]. Padmanaban et al. describe an approximately 9 x 4 cm appendix with LAMN that was treated with an open ileocectomy [5]. No further resection was needed after reviewing the pathology. Kehagias et al. reported a case of an 8.3 x 5 x 4 cm LAMN that was managed with an open right hemicolectomy [9]. Soni et al. report a case of an LMAN managed with exploratory laparotomy with appendectomy alone; however, pathology revealed positive margins [1]. The patient ultimately opted for regular surveillance rather than re-operation for a right colectomy.

## Conclusions

This is the case of a 24-year-old female who presented with nonspecific abdominal pain and equivocal findings of appendicitis and PCS. After a multidisciplinary approach to her care, ongoing pain without relief, and no clear source for her pain, the patient underwent a diagnostic laparoscopy that ultimately discovered a LAMN. This case is unique in that it reinforces the need to keep a broad differential when investigating non-specific sources of pain, laparoscopy as a valuable tool to diagnose and treat pain, and the management of a rare entity that can masquerade as appendicitis.
